# Mothers may shape the variations in social organization among gorillas

**DOI:** 10.1098/rsos.160533

**Published:** 2016-10-19

**Authors:** Andrew M. Robbins, Maryke Gray, Thomas Breuer, Marie Manguette, Emma J. Stokes, Prosper Uwingeli, Innocent Mburanumwe, Edwin Kagoda, Martha M. Robbins

**Affiliations:** 1 Max Planck Institute for Evolutionary Anthropology, Deutscher Platz 6, 04103 Leipzig, Germany; 2 Formerly with the International Gorilla Conservation Programme, PO Box 931, Kigali, Rwanda; 3 Wildlife Conservation Society, Global Conservation Program, 2300 Southern Boulevard, Bronx, NY 10460, USA; 4 Mbeli Bai Study, Wildlife Conservation Society—Congo Program, BP 14537 Brazzaville, Republic of Congo; 5 Volcanoes National Park, Rwanda Development Board, PO Box 6239, Gishushu, Kigali, Rwanda; 6 Parc National des Virunga-sud, Institut Congolais pour la Conservation de la Nature, c/o IGCP-DRC, B.P. 137 Gisenyi, Rwanda; 7 Formerly with the Mgahinga Gorilla National Park, Uganda Wildlife Authority, PO Box 3530, Kampala, Uganda

**Keywords:** maternal investment, dispersal, philopatry, multimale groups, life history, human evolution

## Abstract

When mothers continue to support their offspring beyond infancy, they can influence the fitness of those offspring, the strength of social relationships within their groups, and the life-history traits of their species. Using up to 30 years of demographic data from 58 groups of gorillas in two study sites, this study extends such findings by showing that mothers may also contribute to differences in social organization between closely related species. Female mountain gorillas remained with their sons for significantly longer than western gorillas, which may explain why male philopatry and multimale groups are more common among mountain gorillas. The presence of the putative father and other familiar males did not vary significantly between species, and we found only limited support for the socio-ecological theory that the distribution of adult males is influenced by the distribution of females. Within each gorilla species, variations in those distributions may also reflect the different stages in the typical life cycle of a group. Collectively, our results highlight the potentially far-reaching consequences of maternal support that extends beyond infancy, and they illustrate the opportunity to incorporate additional factors into phylogenetic analyses of variations in social organization, including studies of human evolution.

## 1. Introduction

One of the primary goals of behavioural ecology has been to understand the distributions of adult males and females, including the proportion of breeding groups that are one-male versus multimale [1–3]. Socio-ecological theories predict that the number of adult males per group is mainly determined by the spatio-temporal distribution of sexually receptive females, and the number of adult females per group is influenced by predation risks and the distribution of food resources [4–7]. The number of adult females per group may also be influenced by the number of adult males, particularly if the risk of infanticide is lower in multimale groups than in one-male groups [8,9]. The distributions of adult males and females are shaped by the relative costs and benefits of dispersal versus philopatry [10,11].

The costs and benefits of dispersal (relative to philopatry) may depend on support from parents and other relatives that extends beyond infancy [12,13]. For example, a mother may assist her son in agonistic interactions with other group members, and enhance his access to potential mates, which can influence subsequent male dispersal patterns [14–16]. Maternal support of daughters may facilitate the nepotistic female dominance relationships of many cercopithecines, as well as the post-reproductive lifespan of humans [17–19]. Despite imperfect kin discrimination mechanisms, fathers can support their offspring in agonistic interactions, and provide protection against infanticide and predators [20,21]. Maturing male siblings may emigrate together to maintain coalition partners and reduce the costs of dispersal [22,23]. Dispersal can reduce inbreeding and increase inclusive fitness by reducing competition among kin [24–26]. Life-history parameters can affect the probability that individuals will reach adulthood with their parents and other relatives, but further study is needed to more fully understand the implications for differences in dispersal patterns and social organization among species [27,28].

This paper examines the variations in social organization between/within populations of mountain gorillas (*Gorilla beringei beringei*) and western lowland gorillas (*Gorilla gorilla gorilla*), including the potential influence of parents and other relatives. Gorillas are interesting species for studies of social organization because they represent an important model for understanding the evolution of human behaviour [29–32]. Gorillas are also interesting because both sexes may disperse or remain philopatric, thereby playing a direct role in shaping their group compositions [33–35]. Natal and secondary dispersal are both common for females, who transfer directly to a solitary male or to another group [35–37]. By contrast, adult males (silverbacks) typically become solitary when they emigrate, and they rarely immigrate into breeding groups [34,38,39]. A one-male group is formed when females transfer to a solitary male, and it can become an (age-graded) multimale group when their male offspring reach adulthood [2,40]. A multimale group may become one-male if it fissions, if a subordinate silverback emigrates, or if a silverback dies. When the dominant silverback dies in a multimale breeding group of mountain gorillas, a subordinate silverback inherits the group, and the immature males remain in the group [40,41]. When the dominant silverback dies in a one-male group, the group typically disintegrates, and its immature males may join non-breeding groups [35,42]. If a high proportion of males reach adulthood in non-breeding groups, then the proportion of breeding groups that contain multiple adult males may be correspondingly low.

Approximately 40% of mountain gorilla groups are multimale, versus only 5% for western gorillas, so socio-ecological theories predict that mountain gorilla groups will contain more adult females [43,44]. The largest groups are typically found with mountain gorillas (as expected), but the median group size has not differed significantly among gorilla species [45]. The socio-ecological predictions may be weakened for gorillas because silverbacks rarely immigrate into breeding groups, which limits the potential for the distribution of males to adjust to changes in the distribution of females [38,40].

Less than half of male mountain gorillas disperse upon reaching adulthood, which is significantly lower than the nearly universal dispersal of male western gorillas [38,39]. Those results are consistent with the higher proportion of multimale groups among mountain gorillas, because male philopatry is the primary mechanism for breeding groups to become multimale [40]. Studies of male mountain gorillas have provided only limited support for socio-ecological expectations, however, because the probability of dispersal has not been consistently correlated with the number of adult females or the adult sex ratio in the group [38,40] (but see [46]). Instead, the probability of dispersal was significantly lower if the mother of the potential emigrant was still in the group [38]. Philopatric males had significantly higher copulation rates than males who ultimately emigrated, which suggested that staying in close proximity to their mother could give males more access to other adult females [38]. If the presence of the mother can influence the dispersal patterns and social organization of mountain gorillas, then does it also account for the differences between mountain gorillas versus western gorillas?

In addition to facilitating relationships with other adult females, the mother could help her sons to develop a stronger relationship with the dominant silverback [38]. Those relationships may also be stronger if the sons are with a ‘familiar’ dominant silverback such as their putative father, especially if such familiarity is used as proxy for kin discrimination [47,48]. Silverbacks are considered ‘familiar’ if they were in the group when the potential emigrant was an infant (even if they were not silverbacks at the time). In other words, the potential emigrate grew up with the familiar males, who may include his brothers, half-brothers and his putative father. We define the putative father as the silverback who was dominant when the potential emigrant was first observed as an infant. Dominant silverback mountain gorillas were in close proximity with philopatric subordinates more frequently than with the males who subsequently emigrated, so tolerant relationships could increase the probability for groups to remain multimale [38]. Further study is needed to compare the probabilities for males in each gorilla species to reach adulthood with a dominant silverback who is their putative father or another familiar male.

To examine whether mothers may influence the variations in social organization among mountain gorillas and western gorillas, we compared the probabilities for males in each species to reach adulthood in the same group as their mother. If mothers account for the higher proportion of male philopatry and multimale groups among mountain gorillas, then we expect male western gorillas to have a significantly lower probability of reaching adulthood with their mother. We performed similar comparisons of the probabilities for males to reach adulthood with a dominant silverback who is their putative father or another familiar male. To examine the potential consequences when the dominant silverback dies, we compared the probability for males in each species to reach adulthood in a breeding group (versus a non-breeding group or solitary). To re-examine the socio-ecological theories that the distribution of males is influenced by the distribution of females, we compared the average number of adult females in breeding groups of mountain gorillas versus western gorillas, as well as the average number of adult females in one-male versus multimale groups within each population. Based on the socio-ecological theories, we would expect mountain gorilla groups to contain more adult females than western gorillas, and we expect multimale groups to contain more adult females than one-male groups. We discuss these results within the context of the socio-ecological theories and other potential explanations for the variations in social organization among gorillas and other species, including the development of male philopatry among Homininae.

## 2. Material and methods

Demographic data for mountain gorillas were obtained from the long-term records of the International Gorilla Conservation Programme, the Rwanda Development Board, the Institut Congolais pour la Conservation de la Nature, and the Uganda Wildlife Authority. Mountain gorillas were monitored in 19 groups throughout the Virunga volcano region from June 1979 to April 2010 [41,49]. Data for western gorillas were obtained from the long-term records at Mbeli Bai, a swampy forest clearing in the Nouabalé-Ndoki National Park, Republic of Congo [35,50–52]. Western gorillas were monitored in 39 groups from February 1995 to December 2014. See electronic supplementary material, §S1 for more details about the study sites.

We used a mixed effect Cox model [53] to compare the probabilities for male mountain gorillas and western gorillas to reach adulthood in the same group as their mother. The analysis used a separate data point for each immature male who was observed since infancy (106 mountain gorillas and 96 western gorillas). The predictor variable was the gorilla species. The random effect variables were the identity of the mother and the group where the male was first observed. The response variable was the ‘normalized’ age of the immature males, which we defined as their actual age divided by the age when males were considered adults. The normalized ages enabled us to adjust for differences in the age when mountain gorillas and western gorillas reach adulthood (12 versus 14 years; see electronic supplementary material, §S2 for more life-history differences). Uncensored data points equalled the normalized age when males were separated from their mother, such as when the mother died or transferred to another group. Data points were censored when males reached adulthood with their mother, or at the normalized age when they were no longer observed, such as when they disappeared or the study ended. We used similar Cox models to calculate the probability that an immature male would remain in a group where the dominant silverback was his putative father or any familiar male. See electronic supplementary material, §S3 for more probabilities to remain with potential relatives.

We used a generalized linear mixed model (GLMM, [54]) to compare the probability that males reached adulthood in a breeding group (versus a non-breeding group or solitary). The analysis used one data point for each male who reached adulthood during the study. The response variable equalled ‘1’ if the male reached adulthood in a breeding group, and ‘0’ if he did not. The predictor variable was the gorilla species. The model included the group ID as a random effect variable to control for multiple data points from the same groups. The model was run with a binomial error structure and logit link. See electronic supplementary material, §S4 for more details about the distribution of adult males in each species.

We ran an analysis of variance (ANOVA, [55]) to test whether the number of adult females per group is larger for mountain gorillas than western gorillas. The analysis used one data point for each breeding group in each study site. The predictor variable was the gorilla species. The response variable was the average number of adult females in the group. The average number of adult females in each group was calculated from its composition on the first day of each month when it was observed as a breeding group. To avoid excessive influence from groups with brief observations, each data point was weighted according to the number of months that the group was observed as a breeding group. The reported mean and standard deviation for the number of adult females were also weighted according to the number of months that the group was observed as a breeding group.

We used linear mixed models (LMM, [54]) to test whether the number of adult females per group is larger for multimale groups than one-male groups. The LMM used one data point for each group in each category (one-male versus multimale). The predictor variable was the group category. Some groups were observed in both categories, so we included the group ID as a random effect variable to control for multiple data points from the same group. The response variable was the average number of adult females while the group was in the category. The reported mean and standard deviation for the number of adult females were weighted according to the number of months that the group was observed in the category.

The Cox models were run using the ‘coxme’ function in R, and the ANOVA was performed with the ‘lm’ function (R Core Team 2016). We used the ‘lmer’ function in the ‘lme4’ package for the LMM and GLMM. To determine the *p*-values for the LMM and GLMM, we compared each full model with a reduced model in which the predictor variable had been removed.

## 3. Results

**Figure 1.**
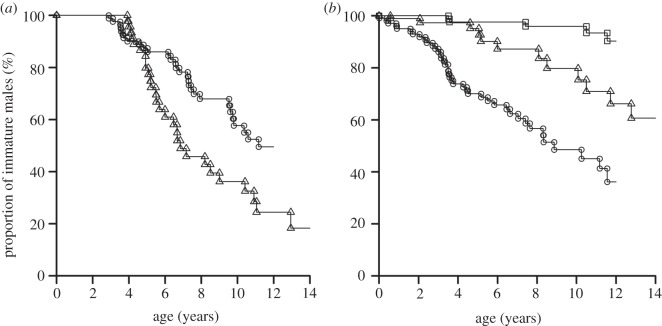
Probability for immature male mountain gorillas (circles) and western gorillas (triangles) to remain with their mother (*a*), or with the dominant silverback who was their putative father (*b*). In (*b*), squares show the probability for immature male mountain gorillas to remain with a dominant silverback who was ‘familiar’. With western gorillas, the results for familiar dominant silverbacks were identical to the results for putative fathers. Symbols are shown at the age of each censored data point, when an immature male was no longer with his potential relative.

Male mountain gorillas had a 50% probability of reaching adulthood in a group with their mother, which is significantly higher than 18% for western gorillas (figure 1*a*; *z* = 3.3, *p* < 0.001). Of the 29 cases when an immature male mountain gorilla was separated from his mother, 45% occurred when the mother transferred to a known destination, 38% occurred when the mother died, 7% occurred when the mother disappeared, another 7% occurred during a group fission, and one case (3%) occurred when the male uncharacteristically transferred to another group (table 1). Transfers by the mother were also the primary cause for separations among western gorillas, but statistical comparisons of female dispersal in each population are confounded by unexplained disappearances (i.e. it is unknown whether some mothers had died or dispersed).

**Table 1.** Causes for male gorillas to be separated from their mother, and to reach adulthood in a group where the dominant male was not their putative father or another familiar male. For example, in 45% of the 29 cases when a male mountain gorilla was separated from his mother, the mother had dispersed from the group.

species	mountain gorillas	western gorillas	mountain gorillas	western gorillas	mountain gorillas	western gorillas
potential relative	mother	mother	putative father	putative father	familiar male	familiar male
*cause of separation*						
adult dispersal	45%	50%				
adult death	38%		58%	100%	60%	100%
adult disappearance	7%	39%			20%	
group fission	7%		18%			
group disintegration		11%				
dominance usurpation			23%			
immature dispersal	3%		3%		20%	
total separations	29	28	40	12	5	12

Male mountain gorillas had a 36% probability of reaching adulthood in a group where their putative father was still the dominant silverback, which is not significantly different from 61% for western gorillas (figure 1*b*; *z* = 0.53, *p* = 0.59). In all 12 cases, when subordinate male western gorillas reached adulthood with a dominant silverback who was not their putative father, the putative father had died (table 1). None of those dominant silverbacks was replaced by another familiar male, so the probabilities for immature male western gorillas to remain with a familiar dominant silverback were the same as the probabilities to remain with their putative father (figure 1*b*).

Male mountain gorillas had a 90% probability of reaching adulthood in a group where the dominant silverback was a familiar male, which is not significantly higher than 61% for western gorillas (figure 1*b*; *z* = 1.7, *p* = 0.095). Of the 40 cases when subordinate male mountain gorillas reached adulthood with a dominant silverback who was not their putative father, he was replaced by another familiar male in 35 cases (88%). The probability of having the putative father replaced with another familiar dominant silverback was significantly higher for mountain gorillas than for western gorillas (88% versus 0%, Fisher exact test, *p* < 0.001).

Only 46% of 54 male western gorillas were in a breeding group when they reached adulthood, which is significantly lower than 83% of 47 male mountain gorillas (*χ*^2^ = 5.3, d.f. = 1, *p* = 0.021). Immigration was the main pathway into non-breeding groups for immature male western gorillas, as expected when one-male breeding groups disintegrate after the death of the dominant silverback. See electronic supplementary material, §S5 for more details about the typical life cycle of gorilla groups.

Mountain gorilla groups contained a weighted average of 5.1 ± 2.7 adult females, which is significantly higher than 3.6 ± 1.5 females for western gorillas (*R*^2^ = 11.6%, *F*_56,1_ = 7.4, *p* = 0.0087). Among mountain gorillas, the weighted average number of adult females was 5.3 ± 3.2 for multimale groups, which is not significantly higher than 4.9 ± 2.6 for one-male groups (*χ*^2^ = 1.8, d.f. = 1, *p* = 0.18). Among western gorillas, the weighted average number of adult females was 1.6 ± 0.67 for multimale groups, which is significantly lower than 3.8 ± 1.5 for one-male groups (*χ*^2^ = 10.2, d.f. = 1, *p* < 0.001). The results for western gorillas are in the opposite direction of expectations, and they arose because groups typically became multimale when the dominant male was old and many of the females had already left.

## 4. Discussion

Variations in the distributions of adult males and females have been attributed to many factors including the distribution of food and other resources, the degree of reproductive synchrony among females, kin competition and cooperation, predation and infanticide, life-history traits and inbreeding [17,25,56]. This study has found significant evidence for a novel theory about distribution of the males: female mountain gorillas remain with their sons for longer than western gorillas, which may lead to greater male philopatry and a higher proportion of multimale groups among mountain gorillas [38,39,47]. We also found limited support for the socio-ecological theory that the number of adult males per group is influenced by the distribution of adult females [57,58]. Collectively, our results highlight the potentially far-reaching consequences of maternal support that extends beyond infancy, and they illustrate the opportunity to incorporate additional factors into comparative studies of variations in social organization among primates and other species [7,59,60].

### 4.1. Presence of the mother and other potential relatives

The probability for males to reach adulthood in the same group with their mother was significantly higher for mountain gorillas than for western gorillas. Male mountain gorillas are significantly more philopatric when their mother is present, perhaps because her proximity facilitates their ability to develop relationships with other females and with the dominant silverback [38,47]. Similarly, northern muriqui mothers (*Brachyteles hypoxanthus*) may help their sons gain access to other adult females, and chacma baboons mothers (*Papio ursinus*) may promote closer paternal relationships for their sons [15,20]. Maternal support may help male hyenas (*Crocuta crocuta*) to secure more favourable dispersal destinations, whereas the more favourable strategy for many male mountain gorillas seems to be philopatry rather than dispersal [14,46,61]. If the presence of the mother can influence the dispersal patterns and social organization among mountain gorillas, then it could also account for differences between mountain gorillas versus western gorillas. One distinction is that essentially all western male gorillas emigrate, even though some of them reach adulthood in the same group with their mother [39]. Western gorillas may have more diffuse spacing within their groups, so even if a male can remain with his mother, he might not gain much access to other group members [62]. Thus, the western male gorillas do not seem to exhibit conditional dispersal that is observed in mountain gorillas and many other species [63,64].

Owing to the limitations in distinguishing between death and dispersal in this study, it is difficult to determine the proximate cause for the differences in the presence of the mother. Hypothetically, the prolonged presence of mothers among mountain gorillas could indicate that they have lower mortality than female western gorillas, but such results would not be consistent with evidence of a slower life history for western gorillas [65–67]. Instead, the prolonged presence of mothers among mountain gorillas is more likely to indicate that they disperse less frequently than parous western gorillas [35,36,68]. Such a distinction would be consistent with findings that female mountain gorillas have lower dispersal rates from multimale groups than from one-male groups, perhaps due to lower risks of infanticide in multimale groups [37,41,69,70]. If so, then a positive feedback loop may develop that increases the proportion of multimale groups among mountain gorillas: an initial formation of multimale groups could reduce the rate of female dispersal, which should enable more sons to reach adulthood with their mothers, which could lead to greater male philopatry and a higher proportion of multimale groups.

Mountain gorillas and western gorillas did not differ significantly in the probability for males to reach adulthood in a group where the dominant silverback was their putative father or another familiar male. Further study is needed to determine whether such relationships can affect whether male mountain gorillas will emigrate or remain philopatric, but the presence of the actual father has not shown any significant effect on dispersal [38,40,46]. Such relationships do not seem to promote philopatry among western gorillas either, because essentially all subordinate males emigrate, even though 68% of them reached adulthood with their putative father as the dominant silverback [39]. Yet even if familiar dominant silverbacks do not promote philopatry among male western gorillas, the end of familiar relationships can lead to involuntary dispersal as reported for Siberian jays (*Perisoreus infaustus*), lions (*Panthera leo*), snub-nosed langurs (*Rhinopithecus* spp.) and other species [13,71,72]. Involuntary dispersal may reduce the probability that male western gorillas reach adulthood in a breeding group, which was significantly lower than the corresponding probability for mountain gorillas in this study [35,42]. The maturation of natal males is the primary mechanism for multimale breeding groups to form, so involuntary dispersal may help to explain why such groups are less common among western gorillas than mountain gorillas [40,73].

### 4.2. Variations in social organization

Socio-ecological models predict that the distribution of males will reflect the distribution of females, and those predictions have been supported by phylogenetic analyses of primates [6,7,59]. Our comparisons between species were also consistent with such predictions, because mountain gorillas had significantly more adult females per group and a higher proportion of multimale groups than western gorillas. One potential caveat is that the difference between species was relatively small in comparison with the variance within species (*R*^2^ = 11.6%), which could help to explain why previous comparisons have not been statistically significant [43,45]. Furthermore, the socio-ecological theory was not supported by our comparisons of social organization within either species, because multimale groups did not have more adult females than one-male groups. Similarly, other studies have found weaker correlations within populations than between species [5,74,75]. Within such populations, variations in social organization may reflect different stages in the life cycle of groups, rather than differences in male reproductive strategies [51,74,76]. Correlations between the distributions of males versus females may also be weakened within populations with high variability in male quality, high dispersal costs and/or limited ability for males to assess reproductive opportunities [5,70,77]. Further study of those influences within species may help to refine our understanding of variations in social organization among species, especially when male dispersal is limited.

### 4.3. Evolution of Homininae (*Gorilla*, *Pan* and *Homo*)

**Figure 2.**
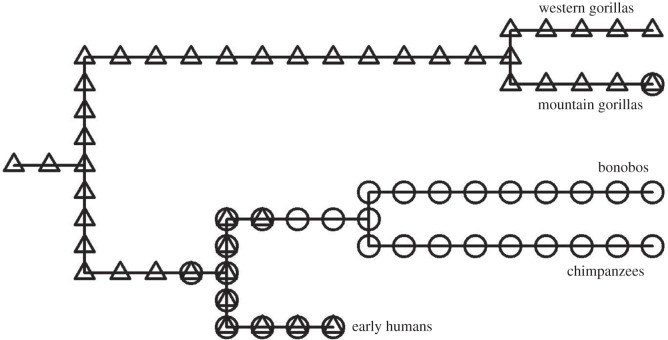
Proposed evolution of male philopatry among Homininae (*Gorilla*, *Pan* and *Homo*). Triangles represent species with predominantly male dispersal, circles indicate philopatry and overlapping symbols reflect a combination of both strategies. The phylogenetic perspective assumes that the trait shared by closely related species was also present in their last common ancestor. The assumption minimizes the number of times that male philopatry would have evolved independently.

The increased proportion of multimale groups has been considered a recent development in the evolutionary history of mountain gorillas, because they lack physiological traits that are typically associated with multimale mating systems [78,79]. If so, then mothers may have facilitated the evolution from the nearly universal dispersal of male western gorillas to the context-dependent philopatry of male mountain gorillas (figure 2). A similar evolution may have gone further for chimpanzees and bonobos, whose multimale social organizations arise from more consistent male philopatry than mountain gorillas [80,81]. Maternal support of sons can extend beyond infancy in both of those *Pan* species, and their parous females disperse less often than gorillas, which increases the probability that the mother will remain present [16,38,82].

The social system of early humans remains unclear, but their degree of male philopatry is often considered greater than western gorillas, and possibly as extreme as the *Pan* species [30,32,83–85]. If so, then male philopatry could have been developing in the last common ancestor of *Pan* and *Homo* when those taxa diverged, even if it subsequently continued further in *Pan* (for additional possibilities, see [31,86]). Thus, the extended maternal support of sons may have contributed to the development of male philopatry for humans and our closest extant relatives.

## Supplementary Material

Presence Of Parents_Supplementary Material.doc

## Data accessibility

Data available from the Dryad Digital Repository: http://dx.doi.org/10.5061/dryad.90nc6 [87]. Additional information is included in the electronic supplementary material.
