# Supplementary material for: Mothers may shape the variations in social organization among gorillas
Source: R Soc Open Sci. 2016 Oct 19;3(10):160533. doi: 10.1098/rsos.160533 (PMC5098995; doi:10.1098/rsos.160533)
Supplement: Presence Of Parents_Supplementary Material.doc [file rsos160533supp1.doc]

Supplementary Material

Section S1. Methods for the supplementary material

Section S2. Life history differences between species

Section S3. Additional probabilities to remain with potential relatives

Section S4. Distribution of adult males in each population

Section S5. Life-cycle of gorilla groups

Section S6. References for the supplementary material

Section S1. Methods for the Supporting Information

*S1.1 Study populations*

Demographic data for the Virunga mountain gorillas were obtained from the long term records of groups that were habituated for tourism [1, 2]. Each of the current habituated groups was continuously monitored by a separate team of observers, who are trained to systematically complete a standardized data sheet during every visit to the gorillas, with special emphasis on rapidly spotting when a gorilla is missing or injured [3]. Each habituated group was generally observed on a daily basis, but monitoring has occasionally been interrupted due to civil unrest, particularly during the mid-1990’s [4].

Data for western gorillas were obtained from observations on a platform in a swampy forest clearing at Mbeli Bai [5-8]. The clearing is typically monitored for ten hours per day, except for two months in 1997 due to civil unrest. The gorilla groups do not visit the clearing every day, so we estimated the dates of birth, death, and dispersal. Some gorillas were observed within 1-2 days after their birth, as confirmed because their group had just been seen without the infant. When gorillas were first observed beyond that age, their birthdates were based on comparisons with other gorillas whose age was already determined [5, 9, 10]. Dispersal dates were typically defined as the midpoint between visits of the groups that the gorilla left/joined [7]. When a gorilla disappeared from the bai, we were generally unable to determine whether it had died or dispersed. Such cases occasionally occur with mountain gorillas too (see Results), and are defined as "disappearances". Dates of disappearance were typically estimated as the midpoint between the last time an individual was observed and the first time that the group was seen without him. If the missing gorilla had been the dominant silverback we assumed that he died, because such males are rarely seen after losing their group [8].

*S1.2 Life history differences between species*

To illustrate how the proportion of multimale groups in each population depends on our criteria for when males reach adulthood, we tallied the age of the oldest subordinate male in each breeding group during each month. For each species, we calculated the proportion of breeding groups with at least one subordinate male who was beyond each age from 10-20 years old. Those results represent what the proportion of multimale groups would be if adulthood was defined to begin at each age.

*S1.3 Probabilities to remain with potential relatives*

We ran a Kaplan Meier analysis to determine how long subordinate male western gorillas *would* have been in a group where the dominant silverback was their putative father, if the subordinate not died or dispersed. The analysis contains one data point for each western gorilla male who was observed since infancy. The response variable was the age of the male when his putative father was last observed as a dominant silverback (regardless of whether the male is still in the group). The data points were censored if the putative father was still dominant at the end of the study, and they were uncensored if the end of his dominance tenure was observed.

*S1.4 Distribution of adult males*

We ran a Fisher exact test to compare the adult sex ratio of mountain gorillas versus western gorillas. The two category variables were the gorilla species (mountain gorillas versus western gorillas) and the sex of each adult (male versus female). The response variable equaled the average number of adult gorillas that were observed in each combination of those two categories. Fisher exact tests were performed using the "fisher.test" function in R (R Core Team 2016).

We ran a similar Fisher exact test to compare the proportion of adult males who were harem holders (versus non-harem holders) in each population. The two category variables were the gorilla species and the status of each adult male (harem holder versus non-harem holder). Again, the response variable equaled the average number of adult gorillas that were observed in each combination of those two categories.

We ran a third Fisher exact test to compare the proportion of non-harem holders who were subordinate males in breeding groups (versus adult males who were solitary or in non-breeding groups) in each population. The two category variables were the gorilla species and the status of each non-harem holder (subordinate adult male versus other non-harem holders).

For all three Fisher exact tests, the average values for mountain gorillas were tallied from the first day of each month since 1998, when the number of study groups increased and the database began to include solitary males. Average values for western gorillas were tallied for the duration of the study (see Methods in the main text). All average values were rounded off to the nearest integer as needed for a Fisher exact test.

To provide another conventional perspective on the distribution of male mountain gorillas, we ran a linear regression model to examine the correlation between the number of adult females versus the total number of adult males (rather than merely considering whether groups were one-male or multimale). The model used one data point for each breeding group. The response variable was the average number of adult males in the group. The predictor variable was the average number of adult females in the group. To avoid excessive influence from groups with brief observations, each data point was weighted according to the number of months that the group was observed.

*S1.5 Life-cycle of gorilla groups*

To compare the proportion of breeding groups that were multimale (versus one-male) in mountain gorillas versus western gorillas, we ran an ANOVA with one data point for each breeding group in each species. The response variable was the proportion of time that each group was multimale. The predictor variable was the gorilla species. To avoid excessive influence from groups with brief observations, each data point was weighted according to the number of months that the breeding group was observed.

We ran a similar ANOVA to compare the proportion of breeding groups that were "old" among mountain gorillas versus western gorillas. Breeding groups were defined as old if they contained a subordinate male who was a blackback or silverback (see more details in section S5 below). The model used one data point for each breeding group in each species. The response variable was the proportion of time that each group was old. The predictor variable was the gorilla species. Again, each data point was weighted according to the number of months that the breeding group was observed.

We ran a third ANOVA to compare the proportion of old groups that were multimale among mountain gorillas versus western gorillas. The model used one data point for each breeding group that was old during the study. The response variable was the proportion of time that each old group was multimale. The predictor variable was the gorilla species. Each data point was weighted according to the number of months that the breeding group was old.

Section S2. Life history differences between species

Western gorillas are considered to have a slower life history than mountain gorillas, as evidenced by their slower rate of maturation and longer interbirth intervals [10-13]. Male mountain gorillas are considered infants until age three, versus age four for western gorillas [10, 14]. Female mountain gorillas are considered adults at age eight, versus ten for western gorillas [10]. Male mountain gorillas are considered adults at age twelve, versus fourteen for western gorillas [10]. The classifications of adulthood for males include "young silverbacks" who have silver coloration on their back but are not yet fully grown [15, 16].

Breeding groups are defined to contain at least one adult female and one adult male. Breeding groups of gorillas are considered "multimale" if they contain more than one adult male. Therefore, the proportion of multimale groups in each population will depend on when males are considered adults during their gradual maturation (Figure S1).

Based on our definitions of adulthood, the 39 western gorilla breeding groups were multimale for 29.5 years and one-male for 234.3 years, so they were multimale for 11% of observations. The 19 mountain gorilla breeding groups were multimale for 98.8 years and one-male for 110.7 years, so they were multimale for 47% of observations.

Male mountain gorillas have been as young as age twelve when they inherited the dominant role, but they are not fully grown until approximately age 16, when they are more capable of becoming dominant through usurpation, group fissions, or emigrating and acquiring females from other groups [1, 17]. If male mountain gorillas were not considered adults until age 16, then 25% of the breeding groups in our study would have been multimale. Only 12% of breeding groups contained a subordinate older than age 20. Most of those males were former dominant silverbacks who typically remain in their group after being usurped (see more details in Section S4.3 below).

Male western gorillas at Mbeli are not fully grown until age 18, when they become start to acquire females after emigrating. Male western gorillas at Mbeli have not inherited the dominant role, or become dominant through usurpation or group fissions [10, 18]. If male western gorillas were not considered adults until age 18, then only 2% of the breeding groups in our study would have been multimale. Thus, the proportion of multimale groups remains higher for mountain gorillas than western gorillas throughout the range of comparable ages for defining male adulthood (Figure S1).

Section S3. Additional probabilities to remain with potential relatives

To extend the analyses in the main text, we explored an idea to consider how long subordinate male western gorillas *would have* stayed in a group where the dominant silverback was their putative father, if the subordinate had not died or dispersed. This approach increases sample sizes for older subordinate males, and it enables us to extend the analysis beyond the age when they disperse. The underlying theory is that western gorillas may disperse because (if they remained philopatric) they would still have a low probability of remaining with their putative father until they were old enough to inherit or usurp their group.

This perspective shows a 60% probability that male western gorillas would have stayed with their putative father until age 16-20, if they had not died or dispersed. The dataset contains ten males who would have reached that age range while their putative father was still the dominant silverback. Those ten males represent a substantial portion of the overall dataset, because only 21 males would have separated from their putative father at any age. The ten males were in four groups where their putative fathers were all still dominant at the end of our observations (Dwayne, Khan, Sangha, and George). Regardless of whether those results are significantly different from mountain gorillas, the 60% probability seems too high to explain why all subordinate western gorillas would need to emigrate. Thus, these results do not support our hypothesis that male western gorillas emigrate because they have a low probability of remaining with their putative father until they are old enough to inherit or usurp their group.

Another perspective would be to consider whether maturing males remain in a group where the dominant silverback is their *actual* father (rather than merely the *putative* father, as examined in the main text). Unfortunately, paternity analyses were not available to consider the actual father in this study, but the analysis in the main text may help to provide some context. Female western gorillas have not been reported to mate with subordinate or extra-group males, and dominant male mountain gorillas have sired as much as 93% of the offspring in their groups, so our results for the putative father should generally reflect the probabilities for remaining with the actual father too [2, 18, 19]. Subordinate silverbacks sire the remaining offspring among mountain gorillas, so in those cases the probability for remaining with the actual father may be similar to our results for familiar males. A recent study of male relationships showed little evidence of paternal kin discrimination among mountain gorillas, and the presence of the actual father has not shown a significant effect on whether males dispersed or remained philopatric [17, 20].

Section S4. Distribution of adult males in each population

*S4.1 Harem holders versus other adult males*

To provide another perspective on the differences in social organization between mountain gorillas versus western gorillas, this section examines the overall distribution of adult males in each population. If male western gorillas are not subordinates in multimale breeding groups, then where are they instead? Hypothetically, they could be harem holders, solitary, in non-breeding groups, or they might not even exist (i.e., the adult sex ratios could differ between populations).

The relative distribution of adult males and females can be expressed by Equation S1:

R = H x p_m_ Equation S1

where R is the adult sex ratio (females:males), H is the average harem size, and p_m_ is the proportion of males who are harem holders [21, 22]. We are loosely defining "harem size" to refer to the number of adult females in any breeding group (including multimale groups), and the "harem holder" is the dominant silverback of the group. The term "non-harem holder" includes subordinate adult males in breeding groups, all adult males in non-breeding groups, and solitary adult males (i.e., all adult males who are not harem holders).

As indicated in the main text, the average harem size of mountain gorilla groups was 5.1 ± 2.7 adult females, which is significantly higher than 3.6 ± 1.5 females for western gorillas (R^2^ = 11.6%, F_56,1_ = 7.4, p = 0.0087). Those results support the socioecological theory that larger harem sizes contribute to a higher proportion of multimale groups among mountain gorillas than western gorillas. As indicated by the R^2^ value, however, only 11.6% of the variance occurred between populations, with the remaining variance occurring among groups within each site. Thus, the harem sizes of western gorillas are often just as large as mountain gorillas.

If the average harem size differs between the two populations, then Equation S1 will not balance unless at least one of the other terms is also different. For example, if both sites had the same sex ratio, then the smaller harem sizes for western gorillas would require a higher proportion of males to be harem holders. From that perspective, smaller harems could lead to fewer multimale groups, simply because fewer males would be non-harem holders (i.e., subordinates). Thus the predicted correlation between harem sizes and multimale groups may partially reflect mathematical constraints instead of socioecological theory.

On average, the adult population of western gorillas contained 48 females and 31 males (R = 1.6), which is not significantly different than 60 females and 27 males (R = 2.3) among mountain gorillas (Fisher exact test, p = 0.33). On average, 42% of the adult male western gorillas were harem-holders, which is essentially identical to 44% of the adult male mountain gorillas (Fisher exact test, p > 0.99). Thus despite the significant difference in harem sizes, neither R nor p_m_ differed significantly among sites, which may reflect the weaker statistical power of the Fisher exact tests. Of those two possibilities, the p-values suggest a stronger probability for western gorillas to have a lower adult sex ratio rather than a higher proportion of harem holders.

Due to the potential mathematical constraints on the correlation between harem size and the proportion of harem holders (Equation S1), a more rigorous test of socioecological theory might be to compare harem size versus the proportion on non-harem holders who are subordinates in breeding groups (rather than in non-breeding groups or solitary). On average, only 11% of the 18 non-harem holders at Mbeli were subordinates in breeding groups, which is significantly lower than 60% of the 15 non-harem holders in the Virungas (Fisher exact test, p = 0.0078). Thus, the distribution of non-harem holders supports the socioecological theory that populations with smaller harem sizes (i.e., western gorillas) have a lower probability for males to be subordinates in multimale groups.

If the two sites have a different proportion of non harem-holders who remain subordinates, then at least one other proportion of non-harem holders must also differ (because the total proportions within each site must add up to 100%). On average, 56% of the non-harem holders at Mbeli were solitary, which is not significantly different from 27% in the Virungas (Fisher exact test, p = 0.16). And 28% of the non-harem holders at Mbeli were in non-breeding groups, which is not significantly different from 13% in the Virungas (Fisher exact test, p = 0.41). Thus, despite the significant difference in the proportions of subordinates, neither of the other two types of non-harem holders differed significantly among sites. The two species differ by a factor of two in both comparisons, so the lack of statistical significance may again reflect insufficient power of the Fisher exact tests. Of those two possibilities, the p-values suggest a stronger probability for western gorillas to have a higher proportion of solitary males, which are more common than males in non-breeding groups in both populations.

The estimates of solitary males (a.k.a. "lone silverbacks" or "LSB") in the Virungas should be considered tentative because the habituation for ecotourism places more emphasis on groups. For comparison purposes, we considered two other estimates for the number of LSB in the Virunga study population:

- Firstly, we compared the habituated study population with the overall gorilla population in the Virungas. The habituated tourist groups have accounted for 46‑48% of the gorillas and 41-44% of the groups in the past three censuses of the entire population (average = 44.5% ± 2.6%). Those three censuses have found an average of 11.7 ± 2.1 LSB, so if the gorillas habituated for tourism accounted for 44.5% of those LSB, then they would have an average of 5.2 LSB per year.
- Secondly, we compared the number of solitary males with the number of emigrants in each study population. The habituated tourist groups had an average of 0.8 males emigrating to become solitary per year, versus 3.1 emigrants per year at Mbeli. The Mbeli study has tracked an average of 10.1 LSB per year, so if the ratio of emigrants : LSB were the same at both sites, then the mountain gorillas study would have an average of 2.7 LSB per year.

Our conclusions in this section would not change if we assumed that the mountain gorilla study had 2.7 or 5.6 LSB per year, instead of the observed value of 3.6 LSB per year.

*S4.2 Additional comparisons between species*

As indicated in Section S4.1 (above), the harem sizes of western gorillas are often just as large as mountain gorillas, even though the difference was statistically significant. Adult male gorillas rarely immigrate into breeding groups, however, so their distribution primarily depends on whether they emigrate or remain philopatric. Thus, when comparing the harem sizes between species, it might be more appropriate to focus on groups that contain potential emigrant males (i.e., compare only the multimale groups, rather than all groups).

Multimale groups of mountain gorillas contained an average of 5.3 ± 3.2 adult females, which is significantly higher than 1.6 ± 0.67 females for multimale groups of western gorillas (R^2^ = 26.5%, F_26,1_ = 9.4, p = 0.0050). The *maximum* number of adult females for a multimale group of western gorillas was four, which is less than the *average* value for multimale groups of mountain gorillas. Furthermore, many of the multimale western gorilla groups had an old dominant silverback who was losing his females. The potential benefits of philopatry depend on social queueing for the dominant position, so future projections of harem sizes may be more important than the number of adult females when a male reaches adulthood [23-25]. Thus, the difference between species becomes more pronounced when the analysis is limited to groups that contained potential emigrants.

*S4.3 Additional perspectives on mountain gorillas*

As stated in the main text, multimale groups of mountain gorillas contained an average of 5.3 ± 3.2 adult females, which is not significantly higher than 4.9 ± 2.6 for one-male groups (Chi-sq = 1.8, df = 1, p = 0.18). Thus, the comparison within the mountain gorilla population did not support the socioecological theory that the distribution of males is influenced by the distribution of females. To provide another perspective on the distribution of male mountain gorillas, we examined the correlation between the number of adult females versus the total number of adult males per group (rather than merely considering whether groups were one-male or multimale). When we aggregated the data into one point for each breeding group, the average number of adult males per group was not significantly correlated with the average number of adult females (R^2^ = 6.4%, F_17,1_ = 1.1, p = 0.30). The R^2^ value indicates that even if the relationship were significant (i.e., with larger sample sizes), the number of adult females would explain less than 10% of the variance in the number of adult males per group. This perspective again suggests that the distribution of adult females is not the primary influence on the distribution of adult males for mountain gorillas.

To examine how usurped (former) dominant silverbacks contribute to the multimale groups among mountain gorillas, we tallied their proportion among all subordinate silverbacks. Five dominant silverbacks have been usurped in the mountain gorilla groups that were habituated for tourism or research, and all of them remained in the group and became subordinates [1, 17, 26, 27]. Collectively, those five males account for approximately 10% of the 350 subordinate silverback-years that have been observed. Thus, usurped silverbacks do not seem to be the primary reason why multimale groups are more common among mountain gorillas than western gorillas.

Section S5. Life-cycle of gorilla groups

*S5.1 Parnell classifications for social organization*

This section examines how the social organization of gorilla populations can depend upon the life-cycle of its groups, such as how they form, how long they last, and how they end. In all gorilla populations, breeding groups can form when a solitary male acquires females. Such groups are not expected to become multimale for at least 12-14 years, when their male offspring begin to reach adulthood (because male immigration into breeding groups is rare). If the group does not last much longer than 12-14 years, then it will typically be a one-male group for most of its history [26]. Thus, the proportion of time that groups are one-male (versus multimale) may partially reflect their duration, relative to the age when males reach adulthood.

Gorilla groups can be classified to reflect the progression in social organization that generally occurs after a solitary male acquires females (adapted from Parnell, 2002).

1. "Nascent" groups contain a silverback and (sub)adult females without offspring, as expected immediately after females transfer to a solitary male. The females may be subadults because dispersal begins before they reach full maturation.
2. "Infant" groups contain the silverback, adult females, and their infants (up to ages 3-4), as expected when the silverback begins to reproduce with the adult females.
3. "Juvenile" groups contain the silverback, adult females, infants, and juveniles (up to ages 6-8), as expected when the adult females begin rearing their second round of offspring with the dominant male.
4. "Sambb" groups contain the silverback, adult females, and immature gorillas that include subadult males (sam = up to ages 8-10) and/or blackbacks (bb = up to ages 12-14). This classification requires that some immature gorillas beyond the juvenile stage must be males, because the subadult females and nulliparous adult females could be immigrants rather than offspring of the silverback.
5. "Multimale" breeding groups contain more than one silverback, adult females, and immature gorillas, as expected when the male offspring begin to reach adulthood.
6. "Senescent" groups contain one or more silverbacks (with the dominant silverback possibly showing signs of advanced age), no with adult females or infants. These non-breeding groups may occur when the adult females transfer to other social units after the weaning or death of their most recent offspring.

In addition to progressing forward through these classifications, a group can also move backward. For example, multimale groups can revert back to one-male "Sambb" groups when a silverback dies or disperses. Therefore, the proportion of time that older groups are multimale versus one-male (Sambb) groups may depend on the frequency at which their blackbacks mature into silverbacks, relative to the frequency at which their silverbacks die or disperse.

To illustrate whether the Parnell classifications generally reflect the progression in social organization among western gorillas and mountain gorillas, we compared the classification of each group at the beginning of each month versus the end of those months (Table S1). In 86% of the months when the classification of a group changed, the group shifted to an adjacent classification within the Parnell progression (squares adjacent to the shaded diagonal in Table S1). In 75% of those shifts to an adjacent classification, the group moved forward through the Parnell classifications (square to the right of the diagonal), and it moved backward in the remaining 25% of those shifts (squares to the left of the diagonal). Thus the Parnell classifications generally seem to describe the temporal changes in social organization among gorillas.

The proportion of breeding groups that are multimale can be expressed as the product of two ratios:

(mmg / bg) = (older / bg) * (mmg / older) Equation S2

Where "mmg" is the number of multimale breeding groups, "bg" is the total number of breeding groups, and "older" is the number of multimale plus Sambb groups.

- If western gorilla groups typically do not last much longer than the age of male maturation, then they may spend most of their history reaching the older classifications, and differences with mountain gorillas could mainly be reflected by the second term in Equation S2 (older/bg).
- Conversely, if groups in both populations typically last much longer than the age of male maturation, then they may spend most of their history toggling back and forth between the multimale and Sambb classifications, and differences between populations could be more apparent in the third term of Equation S2 (mmg/older).

To examine those two hypotheses, we compared how each term in Equation S2 differed between mountain gorillas versus western gorillas:

1. The proportion of all breeding groups that were multimale was 47% during 209.4 group-years observed for mountain gorillas, which is significantly higher than 11% of the 264.8 breeding group-years for western gorillas (R^2^ = 29.6%, F_56,1_ = 23.5, p < 0.001).
2. The proportion of all breeding groups that were old was 78% for mountain gorillas, which is significantly higher than 36% for western gorillas (R^2^ = 31.0%, F_56,1_ = 25.2, p < 0.001).
3. The proportion of old breeding groups that were multimale was 60% during 163.9 group-years observed for mountain gorillas, which is significantly higher than 31% of the 93.8 old breeding group-years for western gorillas (R^2^ = 18.3%, F_35,1_ = 7.8, p = 0.0082).

For both terms on the right side of Equation S2, the ratio for mountain gorillas was twice as high as for western gorillas, so they both make substantial contributions to differences in the term on the left side. Thus the higher proportion of multimale breeding groups among mountain gorillas may arise partially because their groups are more likely to last long enough to become multimale, and because their older groups are more likely to stay multimale.

Collectively, these group dynamics could promote a positive feedback loop that helps to perpetuate a higher proportion of multimale groups among mountain gorillas [26]. Multimale groups can last longer than one-male groups, which typically disintegrate when the dominant silverback dies. Thus, a high proportion of multimale groups can increase the average longevity of groups in a population, which reduces the proportion of time that groups are nascent, infant, or juvenile. Ecological changes are not needed to catalyze such feedback loops, because different types of social organizations can be stable under the same ecological conditions [28, 29]. A substantial proportion of mountain gorillas are still one‑male, which may indicate that the feedback loop is still continuing, or that it is limited by other factors [1, 23].

*S5.2 Males reaching adulthood in non-breeding groups*

A negative feedback loop that limits the proportion of multimale groups may involve the fate of immature males when a one-male group disintegrates after the death of the dominant silverback. If many of those immature males move into non-breeding groups, then the proportion of males who reach adulthood in breeding groups may be correspondingly low, which thereby limits the potential for breeding groups to become multimale. Thus, a low proportion of multimale groups will coincide with a correspondingly high proportion of one-male groups, whose eventual disintegration will further limit the formation of multimale groups. In contrast, when the dominant silverback dies in a multimale group, a subordinate silverback inherits the group, which retains its immature males who can subsequently reach adulthood in a breeding group (thereby helping the group to remain multimale).

As stated in the main text, only 46% of 54 male western gorillas were in a breeding group when they reached age adulthood, which is significantly lower than 83% of 47 male mountain gorillas (Chi-square = 5.3, df = 1, p = 0.021). When the pathway into a non‑breeding group was observed, 86% of the immature male western gorillas had immigrated into the group, and the other 14% were first observed in a breeding group that subsequently lost its females to attrition. Those proportions differ significantly from mountain gorillas, where only 12% of immature males had immigrated into the group, and the other 88% were first observed in a breeding group that subsequently lost its females to attrition (Fisher exact test, p < 0.001). The origin of most immigrants into non-breeding groups is unknown, but immature males rarely emigrate voluntarily, and they often immigrated together with other gorillas, so it seems likely that their former groups had disintegrated when the dominant male died [7, 26, 30]. In contrast, the mountain gorilla non-breeding groups eventually acquired new females and became breeding groups again. Collectively, those results suggest that the potential of forming a multimale breeding group is higher for mountain gorillas than western gorillas.

Section S6. References for the supplementary material

1. Robbins AM, Gray M, Basabose A, Uwingeli P, Mburanumwe I, et al. (2013) Impact of male infanticide on the social structure of mountain gorillas*.* Plos One 8(11).

2. Robbins AM, Gray M, Basabose A, Uwingeli P, Mburanumwe I, et al. (2014) Variance in the reproductive success of dominant male mountain gorillas*.* Primates 55(4): 489-499.

3. Gray M, Kalpers J (2005) Ranger based monitoring in the Virunga-Bwindi region of East-Central Africa: A simple data collection tool for park management*.* Biodiversity and Conservation 14(11): 2723-2741.

4. Robbins MM, Gray M, Fawcett KA, Nutter FB, Uwingeli P, et al. (2011) Extreme Conservation Leads to Recovery of the Virunga Mountain Gorillas*.* Plos One 6(6): e19788.

5. Parnell R (2002) The social structure and behaviour of western lowland gorillas (*Gorilla gorilla gorilla*) at Mbeli Bai, Republic of Congo, Department of Psychology. University of Stirling: Stirling. p. 429.

6. Parnell RJ (2002) Group size and structure in western lowland gorillas (*Gorilla gorilla gorilla*) at Mbeli Bai, Republic of Congo*.* American Journal of Primatology 56(4): 193-206.

7. Stokes EJ, Parnell RJ, Olejniczak C (2003) Female dispersal and reproductive success in wild western lowland gorillas (*Gorilla gorilla gorilla*)*.* Behavioral Ecology and Sociobiology 54(4): 329-339.

8. Breuer T (2008) Male reproductive success in wild western gorillas (*Gorilla gorilla*), Max Planck Institute for Evolutionary Anthropology. University of Leipzig: Leipzig. p. 129.

9. Nowell AA (2005) Behavioural development in wild western lowland gorillas (*Gorilla gorilla gorilla*). University of Liverpool: Liverpool.

10. Breuer T, Hockemba MBN, Olejniczak C, Parnell RJ, Stokes EJ (2009) Physical Maturation, Life-History Classes and Age Estimates of Free-Ranging Western Gorillas-Insights From Mbeli Bai, Republic of Congo*.* American Journal of Primatology 71(2): 106-119.

11. Charnov EL (1993) Life history invariants. Oxford Series in Ecology and Evolution. Oxford: Oxford University Press. 168 p.

12. Robbins MM, Gray M, Kagoda E, Robbins AM (2009) Population dynamics of the Bwindi mountain gorillas*.* Biological Conservation 142(12): 2886-2895.

13. Stoinski TS, Perdue B, Breuer T, Hoff MP (2013) Variability in the Developmental Life History of the Genus Gorilla*.* American Journal of Physical Anthropology 152(2): 165-172.

14. Robbins MM, Robbins AM, Gerald-Steklis N, Steklis HD (2007) Socioecological influences on the reproductive success of female mountain gorillas (*Gorilla beringei beringei*)*.* Behavioral Ecology and Sociobiology 61(6): 919-931.

15. Watts DP (1990) Mountain gorilla life histories, reproductive competition, and sociosexual behavior and some implications for captive husbandry*.* Zoo Biology 9(3): 185-200.

16. Watts DP, Pusey A (1993) Behavior of juvenile and adolescent great apes. Juvenile Primates: life history, development, and behavior. New York: Oxford University Press. pp. 148-167.

17. Stoinski TS, Vecellio V, Ngaboyamahina T, Ndagijimana F, Rosenbaum S, et al. (2009) Proximate factors influencing dispersal decisions in male mountain gorillas, *Gorilla beringei beringei.* Animal Behaviour 77(5): 1155-1164.

18. Breuer T, Robbins AM, Olejniczak C, Parnell RJ, Stokes EJ, et al. (2010) Variance in the male reproductive success of western gorillas: acquiring females is just the beginning*.* Behavioral Ecology and Sociobiology 64(4): 515-528.

19. Vigilant L, Roy J, Bradley BJ, Stoneking CJ, Robbins MM, et al. (2015) Reproductive competition and inbreeding avoidance in a primate species with habitual female dispersal*.* Behavioral Ecology and Sociobiology 69(7): 1163-1172.

20. Rosenbaum S, Hirwa JP, Silk JB, Vigilant L, Stoinski TS (2015) Male rank, not paternity, predicts male-immature relationships in mountain gorillas, *Gorilla beringei beringei.* Animal Behaviour 104: 13-24.

21. Wade MJ, Shuster SM (2004) Sexual selection: harem size and the variance in male reproductive success*.* American Naturalist 164(4): E83-E89.

22. Shuster SM, Wade MJ (2003) Mating systems and strategies. Princeton, NJ: Princeton University Press. 533 p.

23. Robbins AM, Robbins MM (2005) Fitness consequences of dispersal decisions for male mountain gorillas (*Gorilla beringei beringei*)*.* Behavioral Ecology and Sociobiology 58(3): 295-309.

24. Kokko H, Johnstone RA (1999) Social queuing in animal societies: a dynamic model of reproductive skew*.* Proceedings of the Royal Society of London Series B-Biological Sciences 266(1419): 571-578.

25. Ragsdale JE (1999) Reproductive skew theory extended: The effect of resource inheritance on social organization*.* Evolutionary Ecology Research 1(7): 859-874.

26. Robbins MM (1995) A demographic analysis of male life history and social structure of mountain gorillas*.* Behaviour 132: 21-47.

27. Bradley BJ, Robbins MM, Williamson EA, Steklis HD, Steklis NG, et al. (2005) Mountain gorilla tug-of-war: Silverbacks have limited control over reproduction in multimale groups*.* Proceedings of the National Academy of Sciences of the United States of America 102(26): 9418-9423.

28. Chapman CA, Pavelka MSM (2005) Group size in folivorous primates: ecological constraints and the possible influence of social factors*.* Primates 46(1): 1-9.

29. Robbins AM, Robbins MM (2015) Dispersal patterns of females in the genus *Gorilla*. Dispersing Primate Females: Life History and Social Strategies in Male-Philopatric Species. pp. 75-104.

30. Watts DP (1989) Infanticide in mountain gorillas - new cases and a reconsideration of the evidence*.* Ethology 81(1): 1-18.

Figure S1. Proportion of multimale groups among mountain gorillas (triangles) and western gorillas (circles), based on the age males in each species are considered adults. Breeding groups are considered multimale when they contain more than one adult male.

Table S1. Transitions in the social organization of western gorillas (a) and mountain gorillas (b) according to the modified Parnell classifications for nascent (nas), infant (inf), juvenile (juv), Sambb (sambb), multimale (mmg), and senescence (sscc) groups. The rows indicate the group classification at the beginning of a month, and the columns reflect the end of the month. Each cell shows the number of group-months observed. For example, the top-left cell indicates that there were 275 months when a western gorilla group began with a nascent classification, and ended with the same classification. Shaded cells along the diagonal represent months when the group classification did not change. Cells above the diagonal are months when a group moved forward through the classifications, and cells below the diagonal are months when a group moved backward. For example, there were 23 months when a western gorilla group began with a nascent classification, and ended with an infant classification.

a) western gorillas

|  | nas | inf | juv | sambb | mmg | sscc |
| --- | --- | --- | --- | --- | --- | --- |
| nas | 275 | 23 | 1 | 1 | 0 | 0 |
| inf | 6 | 880 | 17 | 0 | 0 | 0 |
| juv | 1 | 2 | 824 | 11 | 0 | 1 |
| sambb | 1 | 0 | 1 | 755 | 14 | 5 |
| mmg | 0 | 0 | 1 | 10 | 343 | 5 |
| sscc | 0 | 0 | 0 | 3 | 6 | 497 |

b) mountain gorillas

|  | nas | inf | juv | sambb | mmg | sscc |
| --- | --- | --- | --- | --- | --- | --- |
| nas | 34 | 2 | 0 | 0 | 0 | 0 |
| inf | 0 | 132 | 5 | 0 | 0 | 0 |
| juv | 0 | 0 | 364 | 9 | 0 | 0 |
| sambb | 0 | 1 | 0 | 767 | 14 | 1 |
| mmg | 0 | 0 | 3 | 6 | 1176 | 2 |
| sscc | 1 | 0 | 0 | 2 | 2 | 221 |
